# The Impact of Selection at the Amino Acid Level on the Usage of Synonymous Codons

**DOI:** 10.1534/g3.116.038125

**Published:** 2017-01-24

**Authors:** Paweł Błażej, Dorota Mackiewicz, Małgorzata Wnętrzak, Paweł Mackiewicz

**Affiliations:** Department of Genomics, Faculty of Biotechnology, University of Wrocław, 50-383, Poland

**Keywords:** amino acid, codon usage, mutation, selection, synonymous codons

## Abstract

There are two main forces that affect usage of synonymous codons: directional mutational pressure and selection. The effectiveness of protein translation is usually considered as the main selectional factor. However, biased codon usage can also be a byproduct of a general selection at the amino acid level interacting with nucleotide replacements. To evaluate the validity and strength of such an effect, we superimposed >3.5 billion unrestricted mutational processes on the selection of nonsynonymous substitutions based on the differences in physicochemical properties of the coded amino acids. Using a modified evolutionary optimization algorithm, we determined the conditions in which the effect on the relative codon usage is maximized. We found that the effect is enhanced by mutational processes generating more adenine and thymine than guanine and cytosine, as well as more purines than pyrimidines. Interestingly, this effect is observed only under an unrestricted model of nucleotide substitution, and disappears when the mutational process is time-reversible. Comparison of the simulation results with data for real protein coding sequences indicates that the impact of selection at the amino acid level on synonymous codon usage cannot be neglected. Furthermore, it can considerably interfere, especially in AT-rich genomes, with other selections on codon usage, *e.g.*, translational efficiency. It may also lead to difficulties in the recognition of other effects influencing codon bias, and an overestimation of protein coding sequences whose codon usage is subjected to adaptational selection.

Redundancy of the genetic code implies that there are more codons than amino acids. Consequently, many amino acids are encoded by more than one codon, which are known as synonymous codons. As a result, some substitutions between these codons are silent and do not change the coded amino acid. For example, in the case of the codons known as fourfold degenerated (4FD), the third codon positions can be freely changed to any nucleotide, without consequences for the coded amino acid, and subsequently for protein composition and function. However, synonymous codons are not used uniformly in real protein coding sequences (*e.g.*, Comeron 2004; Grantham *et al.* 1980; Ikemura 1985; Plotkin and Kudla 2011; Sharp and Li 1986). Such preference of one synonymous codon over others is commonly known as codon usage bias (Sharp and Li 1986). Usage can differ for various genomes and genes within one genome, and even within a single gene.

As far as the evolution of codon bias is concerned, two explanations, which are not mutually exclusive, have been proposed: directional mutations and specific selection (Bulmer 1991; Hershberg and Petrov 2008). From the mutational point of view, GC content is the strongest single determinant of codon usage in genomes (Chen *et al.* 2004; Ermolaeva 2001; Knight *et al.* 2001; Li *et al.* 2015; Muto and Osawa 1987). Thus, in genomes with a high average GC content, the most frequent synonymous codons typically end with guanine or cytosine, whereas, in genomes with a low average GC content, they usually have adenine or thymine in their silent positions. The GC content also fluctuates periodically along vertebrate chromosomes, creating an isochore structure and influencing the local codon usage of genes (Chen *et al.* 2004; Fedorov *et al.* 2002). In prokaryotic genomes, the chromosome-wide codon bias is related to various mutational pressures acting on differently replicating DNA strands, *i.e.*, the leading and lagging strands (*e.g.*, Frank and Lobry 1999; Lobry 1996; Mackiewicz *et al.* 1999b,c; Morton and Morton 2007; Mrazek and Karlin 1998; Rocha *et al.* 1999; Tillier and Collins 2000). As a result, GT-rich codons are usually over-represented in the leading strand genes, whereas AC-rich codons are found in the lagging strand. These codon biases, which are characteristics of genomes, enable the identification of potential genes that have been transferred horizontally (Garcia-Vallve *et al.* 2003).

The bias resulting from mutational effects can be modified by many selectional factors. The first reported influence on the selection on codon bias was based on the observation that highly expressed sequences tend to use generally more frequent codons (*e.g.*, Akashi 2003; Bennetzen and Hall 1982; Clarke 1970; Duret and Mouchiroud 1999; Ghaemmaghami *et al.* 2003; Goetz and Fuglsang 2005; Ikemura 1981, 1985; Kanaya *et al.* 1999; Morton 1998; Rocha 2004). This was interpreted as an adaptation to the effectiveness of the translation process and accuracy of protein synthesis, and is known as codon adaptation or translational selection on codon usage. In addition, a substantial coincidence between gene copy number and the frequency of codons with a concentration of tRNA isoacceptors in their complementary anticodons was detected. The abundant tRNA isoacceptors, through their more fluent recognition of frequently used codons, enable the processivity of translational elongation (Kanaya *et al.* 1999; Xia 1998). Accordingly, there is a significant positive correlation between gene expression level and codon bias, and, likewise, a negative correlation between gene expression level and the rate of synonymous substitutions between compared sequences (Eyre-Walker and Bulmer 1995; Sharp and Li 1987). The selection of synonymous codon usage can also result from selection for translational accuracy to reduce the costs of both missense and nonsense errors (Stoletzki and Eyre-Walker 2007). The effectiveness of translation is also enhanced by clustering some synonymous codons in highly expressed genes, which is called codon co-occurrence bias (Cannarrozzi *et al.* 2010; Shao *et al.* 2012; Zhang *et al.* 2013).

However, the analysis of many genomes has revealed that there is a fraction of genes that show no evidence for translational selection linked to codon usage (Carbone and Madden 2005; dos Reis *et al.* 2004; Sharp *et al.* 2005). This observation is not supported by recent, multi-genome, studies indicating that the translational selection for codon usage seems universal, at least in prokaryotes (Hershberg and Petrov 2008; Supek *et al.* 2010) and plastids (Suzuki and Morton 2016). On the other hand, recently developed techniques measuring endogenous expression have shown that it is the initiation rather than the elongation process that limits the rate of protein production for most endogenous genes (Ingolia 2014; Ingolia *et al.* 2009; Kertesz *et al.* 2010; Tuller *et al.* 2010).

Although translational selection is thought to be the dominant explanation of systematic variation in codon usage among genes (Chaney and Clark 2015; Plotkin and Kudla 2011; Quax *et al.* 2015), several other factors related to codon bias have been put forward. One such factor is the formation of the functional native structure of proteins, which is realized by the preference of common codons in regions critical for protein folding and structure (Oresic and Shalloway 1998; Pechmann and Frydman 2013; Thanaraj and Argos 1996; Zhou *et al.* 2009). Furthermore, bias in synonymous codon usage within the coding sequence is also thought to be an additional layer of information influencing the stability of mRNA structure (Bartoszewski *et al.* 2010; Lazrak *et al.* 2013), mRNA half-life (Presnyak *et al.* 2015), and the effectiveness of transcription (Xia 1996).

It was initially postulated that enrichment of the 5′ end of coding sequences in rare codons is intended to create a ramp at the 5′ end that prevents ribosome traffic jams further down the length of the mRNA, and increases translational efficiency (Tuller *et al.* 2010). Other authors proposed that rare codons cause a translational pause, which helps targeting and export of secreted proteins (Clarke and Clark 2010; Zalucki *et al.* 2009). The ramp concept was revised in further studies showing that the reduced formation of stable mRNA structure is rather responsible for the higher translation rate (Bentele *et al.* 2013; Goodman *et al.* 2013; Kudla *et al.* 2009), whereas a computational model predicted that this ramp is caused by rapid initiation of short genes rather than rare codons at the 5′ end of transcripts (Shah *et al.* 2013).

The presence of many selective constraints on codon usage has consequences for the slower synonymous substitution rate of genes subjected to these selections, as demonstrated by the inverse correlation between the rate and the degree of codon adaptation (Morton *et al.* 2002; Sharp and Li 1986; Sharp *et al.* 1989; Shields *et al.* 1988; Sorhannus and Fox 1999). An understanding of the rules in codon usage is also important in order to better optimize heterologous gene expression (Gustafsson *et al.* 2004), produce vaccines with attenuated viruses (Coleman *et al.* 2008), or find association of diseases with synonymous single nucleotide polymorphism (Daidone *et al.* 2011; Kimchi-Sarfaty *et al.* 2011; Sauna and Kimchi-Sarfaty 2011). Therefore, it is still important to better recognize mechanisms that induce codon usage biases in nature, to understand how the codon landscape evolves with time, and to search for other factors affecting codon bias.

In his seminal work, Morton (2001) postulated another important factor influencing synonymous codon usage. Interestingly, it is not related to direct adaptational selection of codon usage, but results only from a general selection of protein coding sequences at the amino acid level. His analyses showed that the composition of the silent sites of codons deviates from the composition of noncoding (neutral) sites even in the absence of selective differences between synonymous codons. This results from various probabilities of fixation of codon replacements. Morton nicely demonstrated that, after considering this type of selection, there are far fewer genes with codon adaptation bias than previously thought. This implies that selection acting on codon usage associated with translational efficiency may be overestimated. However, the study considered only four selected mutational processes, generating equal frequencies of complementary nucleotides.

To further explore this subject, we created a mutation–selection model that includes the most general and unrestricted model of nucleotide substitutions, and examines a large number of possible mutation processes, generating almost 90,000 stationary distributions of codons. We applied an adapted version of the evolutionary optimization algorithm to find conditions in which mutation processes, together with selection at the amino acid level, maximizes the degree of codon bias (defined as deviation from uniform codon usage). The results demonstrate that the effect under study cannot be neglected.

## Methods

### Overview

One of the best ways to assess the influence of selection, at the amino acid level, on synonymous codon usage is to compare the codon frequency resulting from selection with the expected frequency without this constraint. To achieve that, we constructed a mutation–selection model similar to that of Morton (2001). This model is based on the theory of homogeneous and continuous-time Markov processes. In contrast to Morton (2001), we examined the most general nucleotide substitution model, which was superimposed on the codon selection process associated with the physicochemical properties of coded amino acids. Moreover, we tested almost 90,000 stationary distributions of codons, which corresponds to the mutational process. The effect of selection based on differences between relative codon usage was measured before and after the applied selection. Since a given nucleotide stationary distribution can be realized by many Markov processes, we applied an evolutionary based optimization algorithm in order to find conditions in which the differences between relative codon usage are maximized. As a result, we were able to determine the effect produced by the model with amino acid selection on synonymous sites. In the following sections, we describe in detail the stages of this approach, which is presented in Supplemental Material, Figure S1. Finally, the theoretical calculations were compared with results provided by bacterial genome analyses.

### Mutation process

To model the process of pure mutational pressure expressed by single nucleotide substitutions, we applied a homogeneous, stationary, and continuous-time Markov process. The process is described by a substitution rate matrix, *Q*, and stationary distribution of nucleotides, *π*. This approach is most commonly used in the description of DNA sequence evolution (Yang 2006). Here, we used the most general unrestricted model of nucleotide substitution, called UNREST (Table 1) (Yang 1994).

**Table 1:** Substitution rate matrix *Q* for the unrestricted model of nucleotide substitutions (UNREST). The diagonals of *Q* are determined by the requirement that each row sum to zero

	A	T	G	C
A	—	$q_{AT}$	$q_{AG}$	$q_{AC}$
T	$q_{TA}$	—	$q_{TG}$	$q_{TC}$
G	$q_{GA}$	$q_{GT}$	—	$q_{GC}$
C	$q_{CA}$	$q_{CT}$	$q_{CG}$	—

The nucleotide stationary distribution $\pi =\left(\pi_{A},\pi_{T},\pi_{G},\pi_{C}\right)$ is given by the set of equations πQ=0 under the constraint $\sum_{i\in \{A,T,G,C\}}\pi_{i}=1.$

The assumption about the stationarity of this process implies immediately the need to determine its exact stationary distribution, which should correspond in this case to the stationary frequency of nucleotides generated by the mutational process. In his work on this topic, Morton (2001) considered only four selected mutational processes, with fixed nucleotide stationary distributions characterized by high A+T content. To formulate more general conclusions, and to assess the influence of selection, at the amino acid level, on various mutational processes, we analyzed 88,560 different nucleotide stationary distributions for the potential processes, which cover various mutational pressures. The frequencies of particular nucleotides range from 0.05 to 0.85, with 0.01 increments. Therefore, they form the set:

$$\Pi =\left\{\pi :\pi =\left(\pi_{A},\pi_{T},\pi_{G},\pi_{C}\right),\;\sum_{i}\pi_{i}=1\right\},$$ (1)

where every π∈Π is chosen according to the following assumptions:

1. $0.05\leq \pi_{i}\mathrm{\leq 0}\mathrm{.85,}i\in \left\{A,T,G,C\right\};$
2. for every π∈Π, the Euclidean distance to the nearest stationary distribution, $\pi^{\prime }\in \Pi$, = 0.02, and the difference between $\pi^{\prime }$ and π in one coordinate = 0.01 or 0.

As a result, we obtained a dense subset of all possible nucleotide stationary distributions.

To find the rates of the matrix *Q* for particular distributions, we rested on the assumption that, for the homogeneous, continuous-time, and stationary Markov process, the following set of equations holds:

πQ=0. (2)

These equations can be reformulated easily into a system of three equations:

$$V\beta^{T}=0,$$ (3)

where:

$$V=\left[\begin{array}{c} -\pi_{A}\\ \pi_{A}\\ 0 \end{array}\begin{array}{c} -\pi_{A}\\ 0\\ \pi_{A} \end{array}\begin{array}{c} -\pi_{A}\\ 0\\ 0 \end{array}\begin{array}{c} \pi_{T}\\ -\pi_{T}\\ 0 \end{array}\begin{array}{c} 0\\ -\pi_{T}\\ \pi_{T} \end{array}\begin{array}{c} 0\\ -\pi_{T}\\ 0 \end{array}\begin{array}{c} \pi_{G}\\ 0\\ -\pi_{G} \end{array}\begin{array}{c} 0\\ \pi_{G}\\ -\pi_{G} \end{array}\begin{array}{c} 0\\ 0\\ -\pi_{G} \end{array}\begin{array}{c} \pi_{C}\\ 0\\ 0 \end{array}\begin{array}{c} 0\\ \pi_{C}\\ 0 \end{array}\begin{array}{c} 0\\ 0\\ -\pi_{C} \end{array}\right]$$

and

$\beta \in R^{\mathrm{12}}$ is composed of 12 substitution rates of matrix *Q*:
$\beta =\left[q_{AT},q_{AG},q_{AC},q_{TA},q_{TG},q_{TC},q_{GA},q_{GT},q_{GC},q_{CA},q_{CT},q_{CG}\right]$ under the constraint:
  $$\forall_{i\neq j}q_{ij}>0,i,j\in \left\{A,T,G,C\right\},$$ (4)

which is necessary to create a homogeneous, continuous-time Markov processes with fixed stationary distribution.

The set of Equation 3 has infinitely many nontrivial solutions. Moreover, each solution can be described by a linear combination of independent vectors, $v_{1},v_{2},\ldots ,v_{9}\in R^{12}$, with coefficients $\beta_{i},i=1,2,\ldots ,9:$

$$\beta =\beta_{1}v_{1}+\beta_{2}v_{2}+\ldots +\beta_{9}v_{9}.$$ (5)

The **β** allows creation of the matrix *Q*, from which we derived a nucleotide transition probability matrix, *P*, by adopting the uniformization method (Jensen 1953; Tijms 2003). Generally, the uniformization procedure is used to transform the original continuous-time Markov process with nonidentical leaving rates into an equivalent of stochastic process, in which the transition epoch is generated by a suitable Poisson process with a fixed rate. Following this method, for a given *Q* with stationary distribution *π*, we could define a transition probability matrix $P=\left(p_{ij}\right),i,j=A,T,G,C$ assuming that:

$$p_{ij}=\{\begin{array}{ll} \frac{q_{ij}}{q}, & i\neq j;\\ 1-\frac{\left|q_{ij}\right|}{q}, & i=j, \end{array}$$ (6)

where $q=\sum_{i\in A,T,G,C}\left|q_{ii}\right|.$ Clearly, *P* is the transition probability matrix describing the Markov chain with stationary distribution *π*, which is the same for the continuous case. Moreover, the sum of all its off-diagonal elements is equal to one. This representation turned out to be very useful in our mutation–selection model because it allowed the construction of quite a large set of possible nucleotide transition probability matrices, *P* (Figure S1), under relatively weak mathematical assumptions.

Obviously, we are interested in a codon substitution process, and, for this reason, we calculated a codon *k* to codon *l* transition probability matrix $P*=\left(p_{k\to l}^{*}\right),$ using the nucleotide transition probability matrix *P* (Figure S1). In the matrix P*, we took into account all independent substitutions between codons resulting from a single nucleotide change. The Markov chain defined by P* is also stationary, with codon stationary distribution *π^cod^*, which is in accordance with the following system of equations:

$$\pi^{cod}\left(I-P*\right)=0.$$ (7)

Moreover, under the assumptions presented above, the stationary relative frequencies of 4FD are determined solely by the stationary distribution π.

### Process of selection

Similarly to Morton (2001), we were interested in a model of sequence evolution that combines mutation and selection at the amino acid level. At the selection stage, we introduced the acceptance matrix, $D=\left(d_{m\to n}\right),$ which contains probabilities that a change of amino acid *m* to amino acid *n* will be “accepted.” All diagonals of matrix *D* are equal to one, which means no selective costs of substitutions between synonymous codons. In this work, we employed the acceptance matrix presented in Morton (2001), which is based on Grantham’s (1974) chemical similarity matrix (Figure S1). Additionally, we took into account two cases involving substitutions to and from stop codons. In the first case, we assumed that such mutations are lethal (SL), and we set the probability of acceptance to zero. In the second case, the probability of acceptance of such substitutions was equal to the minimal probability in the matrix D (SM).

As a consequence, we defined a general model, including the mutation and selection processes, in the same way as in Morton (2001). This model is expounded by a codon to codon transition probability matrix $C=\left(c_{k\to l}\right).$ Furthermore, every codon to codon substitution $c_{k\to l}$ is defined by the following equation:

$$c_{k\to l}=p_{k\to l}^{*}\times d_{m\to n},$$ (8)

where $p_{k\to l}^{*}$is the transition probability between codons *k* and *l*, whereas $d_{m\to n}$ is the probability of accepting a change from amino acid *m* to amino acid *n* coded by codons *k* and *l*, respectively. Obviously, all diagonals in matrix *C* are set to make the rows sum to one. In addition, the Markov chain described by *C* has its own stationary distribution *π^sel^*.

### Measure of selection strength

The strength of selection at the amino acid level, which affects the composition in neutral sites of codons, was assessed for each stationary distribution *π* by the normalized difference between the relative frequency of 4FD codons after selection, and their expected frequency resulting only from a mutation process:

$$F_{\pi |s}=\sum_{i\in A,T,G,C}\frac{\left|\pi_{i}-\frac{\pi_{s_{i}}^{sel}}{\pi_{s}^{sel}}\right|}{\pi_{i}},$$ (9)

where *s* means a group of 4FD codons coding for one amino acid; *s_i_* is a codon from this group, in which a nucleotide *i* occurs at the third position; $\pi_{s}^{sel}=\sum_{i\in A,T,G,C}\pi_{s_{i}}^{sel}$is the stationary frequency of this codon group after selection (*sel*);$\pi_{s_{i}}^{sel}$ is the stationary frequency of codon *s_i_* after selection; *π_i_* is the relative stationary frequency of codon *s_i_* obtained only from the mutation process. We analyzed all five groups of 4FD codons, and calculated the summarized effect of the selection at the amino acid level on these groups for each stationary distribution *π*:

$$F_{\pi }=\sum_{s\in S}F_{\pi |s},$$ (10)

where *S* is the set of all groups of 4FD codons *s*. Clearly, large values of *F_π_* suggest a strong impact of selection on the usage of 4FD codons, whereas values equal to zero indicate a lack of such an effect on the relative frequencies of 4FD codons.

### Simulation procedure

A nucleotide stationary distribution *π* can be realized by many Markov processes described by various substitution matrices, *P_π_*, which can imply differences in stationary frequencies of codons after the selection *π^sel^*, and, consequently, different *F_π_* values. To deal with this problem, we decided to find the probability $P_{\pi }^{\max}$ that maximizes the *F_π_* measure. The maximum value of *F_π_* was denoted by $F_{\pi }^{\max}.$ Consequently, we were able to assess the range of selection strength at the amino acid level on synonymous codon usage for a given nucleotide stationary distribution *π*.

The task of finding $F_{\pi }^{\max}$ is, in fact, an example of a single objective optimization problem, where *F_π_* is a fitness function. Therefore, we decided to use the Evolutionary Strategies (ES) approach (De Jong *et al.* 1997), which is a commonly used technique in optimization problems when the solution is hard to find analytically. For each nucleotide distribution *π*, we ran simulations with a population of 100 random candidate solutions according to ES principles. At the beginning of each simulation run, our candidate solutions were, in fact, substitution rate matrices *Q* selected at random according to the procedure described by Equation 3 and condition (4). In every simulation step, we applied mutation and selection operators. For a given rate matrix (individual), the process of mutation was realized by a random modification of its vector of coefficients $\beta_{i},i=1,2,\ldots ,9$ according to the normal distribution *N*(0,*σ*) (Figure S1). The *σ* parameter was tuned during preliminary simulation tests to obtain a quick convergence to the satisfactory solution. The crossover operator used in this problem was a modified version of the Linear Crossover LBGA (Schlierkamp-voosen and Muhlenbein 1994). This produced an offspring that was a random linear combination of its parents in terms of Equations 3 and 5. Understandably, at the end of these procedures, we checked the quality of newly produced offspring, to find out whether they possess a proper representation, and fulfill condition (4). In the next step, we made transformations of substitution nucleotide matrices *Q* to *P*, and next to substitution codon matrices P* and *C*. This was done according to the procedure described in the previous sections. Therefore, we were able to calculate the codon stationary distribution after selection *π^sel^*, and values of the fitness function *F_π_*. Finally, we used tournament selection as the selection operator. Depending on the assumed fitness function, the algorithm selected individuals (rate matrices) that maximized the measure *F_π_*. The main program was developed by the authors (P.B., D.M. M.W., and P.M) in C++ language. The stationary vectors *π^sel^* were calculated using the Armadillo library (Sanderson 2010).

### Analysis of deviation in codon usage in protein coding sequences

The values determined for the $F_{\pi }^{\max}$ measure were compared with an analogous parameter calculated for 4FD codons, using protein coding sequences from 4879 fully sequenced bacterial genomes, whose sequences and annotations were downloaded from the NCBI database (ftp://ftp.ncbi.nlm.nih.gov/genomes). We examined separately the genes located on the leading and the lagging DNA strands. The boundaries between the DNA strands were determined according to DNA walk methods, using DNA asymmetry parameters, *i.e.*, the differences in complementary nucleotides: [G–C] and [A–T] (Kowalczuk *et al.* 2001; Mackiewicz *et al.* 1999a). For these data, we calculated the summarized deviation from the expectation in the codon usage for all 4FD groups *S*. Clearly, this corresponds to Equations 9 and 10, *i.e.*:

$$F=\sum_{s\in S}f_{s},$$ (11)

where:

$$f_{s}=\sum_{i\in A,T,G,C}\frac{\left|e_{i}-\frac{o_{s_{i}}}{o_{s}}\right|}{e_{i}}$$ (12)

is the normalized difference between the relative frequencies of 4FD codons in one group, and their expected frequencies. Therefore,$o_{s_{i}}$is the observed frequency of a 4FD codon *s_i_*, with a nucleotide *i* at the third position,$o_{s}=\sum_{i\in A,T,G,C}o_{s_{i}}$ is the frequency of all codons in the group, and *e_i_* is the expected frequency established as the average of relative frequencies of all 4FD codons with a nucleotide *i* at the third codon position.

### Data availability

Figure S1 illustrates the procedure leading to the assessment of the selection strength, at the amino acid level, and Figure S2 compares nucleotide substitution probabilities for 5% of top matrices that maximized the values of *F_π_*.

## Results and Discussion

### The summarized effect of selection on all codon groups

In total, we performed 88,560 simulations to find the maximum $F_{\pi }^{\max}$ values for the normalized difference between the relative frequency of 4FD codons after selection on amino acids, and their expected frequency triggered only by a mutation process. This parameter expresses the most extreme impact of selection at the amino acid level on the usage of 4FD codons. This impact can be found for a given mutation process with its specific nucleotide stationary distribution *π*. Selection with lethal stop codons’ substitutions (SL) and the variant with the minimal acceptance probability of such substitutions (SM) were studied separately.

The applied optimization algorithm enabled an effective maximization of the fitness function *F_π_* for all the nucleotide stationary distributions *π* under study. Our results indicate that it is possible to find transition probability matrices $P_{\pi }^{\max}$ for each nucleotide stationary distribution *π*, which maximize the impact of such a selection measured by $F_{\pi }^{\max}.$ Generally, depending on the applied stationary distribution *π*, $F_{\pi }^{\max}$varied from 0.25 to over 9.22 under SL variant, and from 0.26 to over 9.1 under SM variant.

The nucleotide stationary distributions for which the extreme values of $F_{\pi }^{\max}$ were found are presented in Table 2. The findings indicate that the largest impact of selection at the amino acid level on deviations in 4FD codons usage is for mutation processes that generate thymine with high frequency at the expense of guanine and cytosine. The next most frequent nucleotide is adenine. On the other hand, the smallest $F_{\pi }^{\max}$ is for nucleotide distributions with high content of cytosine, and next guanine. To systematically analyze the relationship of $F_{\pi }^{\max}$ to the nucleotide stationary distributions, we carried out additional studies. Since the results for models SL and SM were very similar, we focused on the latter.

**Table 2:** Nucleotide stationary distributions of mutation process for which extreme $F_{\pi }^{max}$values were found

Selection model	$F_{\pi }^{max}$	A	T	G	C
SL	9.22	0.15	0.74	0.05	0.06
	0.25	0.05	0.07	0.13	0.75
SM	9.10	0.19	0.69	0.05	0.07
	0.26	0.05	0.08	0.22	0.65

Models with the selection assuming lethal substitutions involving stop codons (SL), and the variant with the minimal acceptance probability of such substitutions (SM), were considered separately.

**Figure 1:**
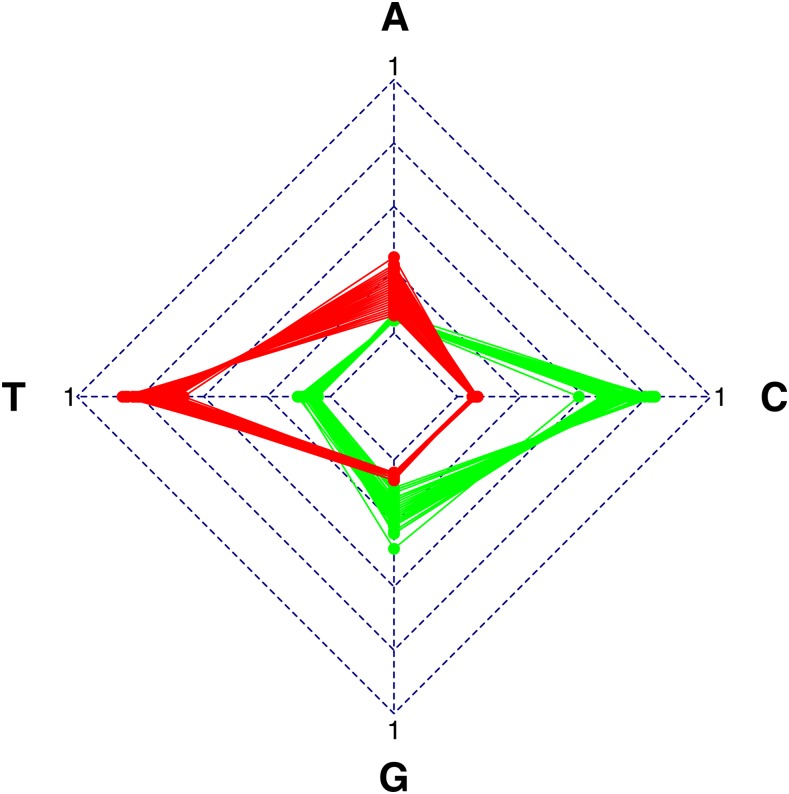
Comparison of two sets of 100 stationary distributions for which $F_{\pi }^{\max}$ (the normalized difference between the relative frequency of 4FD codons after selection on amino acids, and their expected frequency resulting only from a mutation process) takes the highest (red) and the lowest values (green). The $F_{\pi }^{\max}$ is the highest for the distributions with the high frequency of thymine and adenine, respectively, whereas the lowest for the distributions rich in cytosine and guanine, respectively.

The results for extreme values are supported by the radar chart, in which two sets of 100 stationary distributions responsible for the highest and the lowest values of $F_{\pi }^{\max}$ are presented (Figure 1). It can be seen that the $F_{\pi }^{\max}$value is clearly related to the frequency of nucleotides in the stationary distribution. The excess of thymine, and then adenine, leads to the highest values of $F_{\pi }^{\max},$ whereas the lowest values of $F_{\pi }^{\max}$ are observed for the domination of cytosine, and then guanine.

**Figure 2:**
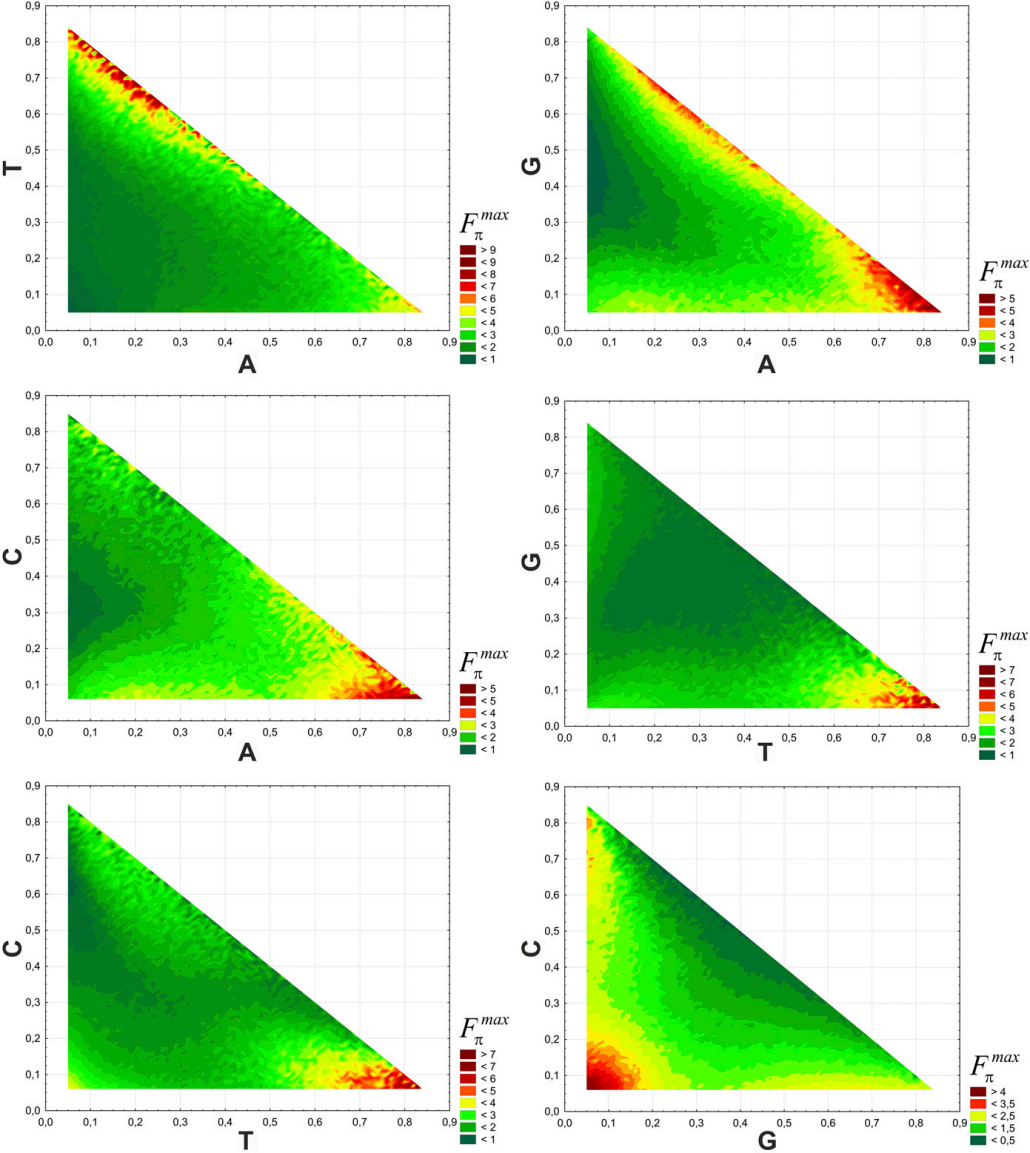
Relationship between the $F_{\pi }^{\max}$ value and combination of two nucleotides presented as colored Wafer maps. The colors correspond to the value of $F_{\pi }^{\max},$ which depends on the frequency of the compared nucleotides. Dark green corresponds the lowest values, and dark brown the highest values of $F_{\pi }^{\max}.$ Its highest values are for the high content of thymine and adenine, with simultaneous decrease in the guanine and cytosine frequency. The lowest values are for the low frequency of A and T, as well as for moderate content of G and C.

In order to study how the possible maximum deviation in the usage of 4FD codons $F_{\pi }^{\max}$ depends on the whole range of nucleotide stationary distributions in the combination of two nucleotides, we made the Wafer map, in which the gradient coloring corresponds to the $F_{\pi }^{\max}$ value (Figure 2). Dark green denotes the lowest values, and dark brown the highest values, of $F_{\pi }^{\max}.$ The relationships are clearly nonlinear. The highest values are observed for distributions with high frequency of thymine, and a substantially smaller contribution of other nucleotides, especially for *π_T_* > 0.6 and *π_A_* in the range from 0 to 0.25, as well as for *π_T_* > 0.7 and *π_G_* < 0.2 or *π_C_* < 0.2. The increase in $F_{\pi }^{\max}$ also correlates with the high frequency of adenine *π_A_* > 0.7, but only together with the decline of guanine and cytosine frequencies to values <0.2. However, there is a growth of $F_{\pi }^{\max}$also for *π_A_* from 0.2 to 0.4 with the excess of guanine in the range 0.5–0.7.

The lowest $F_{\pi }^{\max}$ values are obtained for the substitution matrices generating a low frequency of adenine (*π_A_* < 0.1), with *π_T_* < 0.5, *π_G_* from 0.3 to 0.6 and *π_C_* from 0.2 to 0.4 (Figure 2). $F_{\pi }^{\max}$ has low values also for the frequency of cytosine in the range from 0.45 to 0.65, when the content of thymine is very small (*π_T_* < 0.1), and for guanine from 0.4 to 0.6, when thymine shows a moderate content of 0.3–0.5. The values of $F_{\pi }^{\max}$are also low for *π_G_* from 0.2 to 0.5, and *π_C_* from 0.4 to 0.7.

We also analyzed the impact of stationary frequencies of particular nucleotides on the values of$F_{\pi }^{\max}.$ Therefore, we created the sets $\Pi^{A},\Pi^{T},\Pi^{G},\Pi^{C},$ which are defined in the following way:

$$\Pi^{A}=\underset{k\in K}{\cup }\Pi_{k}^{A},$$

where $\Pi_{k}^{A}=\left\{\pi :\pi \in \Pi \wedge \pi_{A}=k\right\}$ and k∈K={0.05,0.06,…,0.84,0.85}. For example, $\Pi_{0.05}^{A}$ is the set of all stationary distributions, *π*, when the frequency of adenine is 0.05, *i.e.*$\pi_{A}=0.05,$ whereas frequencies of other nucleotides sum up to 0.95, *i.e.*, $\sum_{i\in T,G,C}\pi_{i}=1-\pi_{A}=0.95.$ The sets $\Pi^{T},\Pi^{G},\text{and }\Pi^{C}$ were described in the same way.

Following this approach, we decided to calculate $me\left(F_{\pi }^{\max}\right),$ *i.e.*, the median value of $F_{\pi }^{\max}$ for every nucleotide N=A,T,G,or C, and the stationary distribution $\pi \in \Pi_{k}^{N}$ separately. The main reason for using the median can be explained by the fact that it is an estimator of location parameter that is most resistant to outliers. Therefore, it is a useful and stable measure with which to detect general tendencies in large data sets. In addition, $me\left(F_{\pi }^{\max}\right)$ for $\pi \in \Pi_{k}^{N}$ is a function of k∈K for every N=A,T,G,or C. In other words, the median was calculated from $F_{\pi }^{\max}$ values that were derived from substitution models generating nucleotide stationary distributions with the fixed frequency of one nucleotide and random frequencies of others.

**Figure 3:**
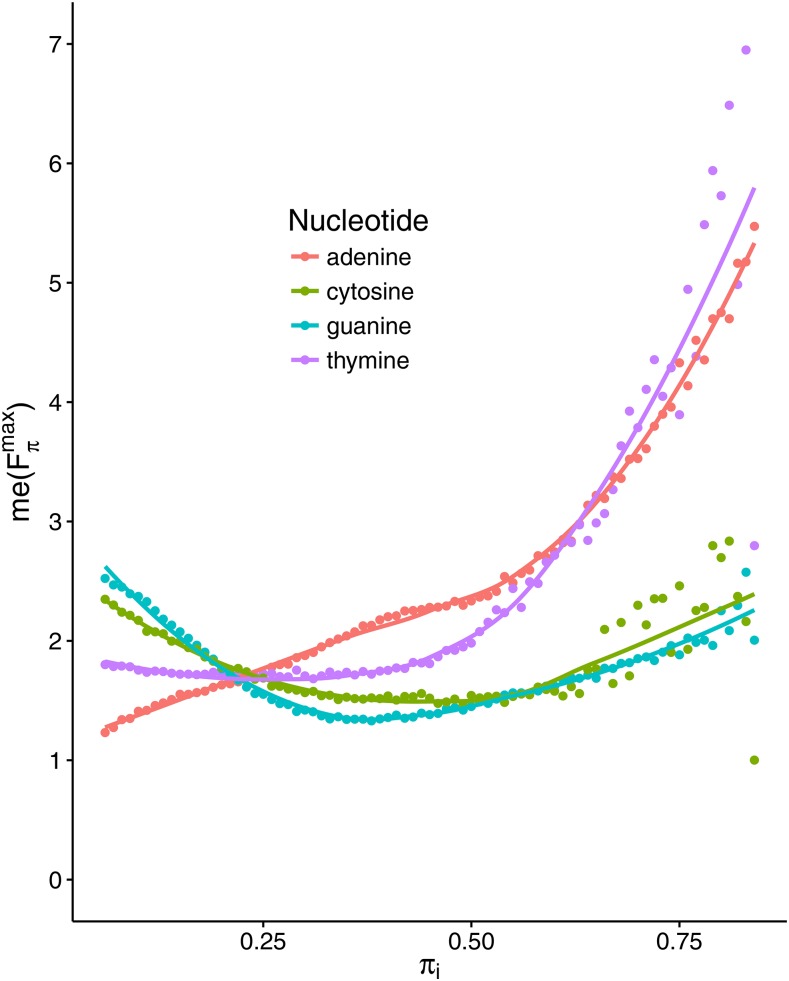
Dependence of median value of $F_{\pi }^{\max},$ *i.e.*, $me\left(F_{\pi }^{\max}\right)$ on stationary frequencies of four nucleotides *π*. The median was calculated from $F_{\pi }^{\max}$ values that were derived from substitution models generating nucleotide stationary distributions, with the given fixed frequency of one nucleotide *π_i_* and random frequencies of others. The dots represent exact values of $me\left(F_{\pi }^{\max}\right),$ whereas lines are the best approximation based on generalized additive models with integrated smoothness estimation. The $me\left(F_{\pi }^{\max}\right)$ depends nonlinearly on the stationary distribution of particular nucleotides. Its strongest increase is for the growth of A and T.

In Figure 3, we illustrate the dependence of the median value of $F_{\pi }^{\max}$ on the stationary frequencies of four nucleotides. Interestingly, the relationships are not linear, and $me\left(F_{\pi }^{\max}\right)$ shows a similar course for complementary nucleotides, especially for guanine and cytosine. The median value of $F_{\pi }^{\max}$ starts from a relatively high value for small frequencies of G and C, and decreases gradually with their growth, reaching a minimum for their frequencies ∼0.36. After that, the median rises steadily, reaching its maximum for the highest frequencies of G and C. However, for the adenine frequency, $me\left(F_{\pi }^{\max}\right)$ grows steadily to 0.6, and then increases rapidly for the highest frequencies. In the case of thymine, the median remains quite constant until it reaches 0.4, and then also quickly increases. The median values of $F_{\pi }^{\max}$ for the fixed frequencies of G and C are generally lower than for A and T, with the exception of the frequency of G and C <0.2.

**Figure 4:**
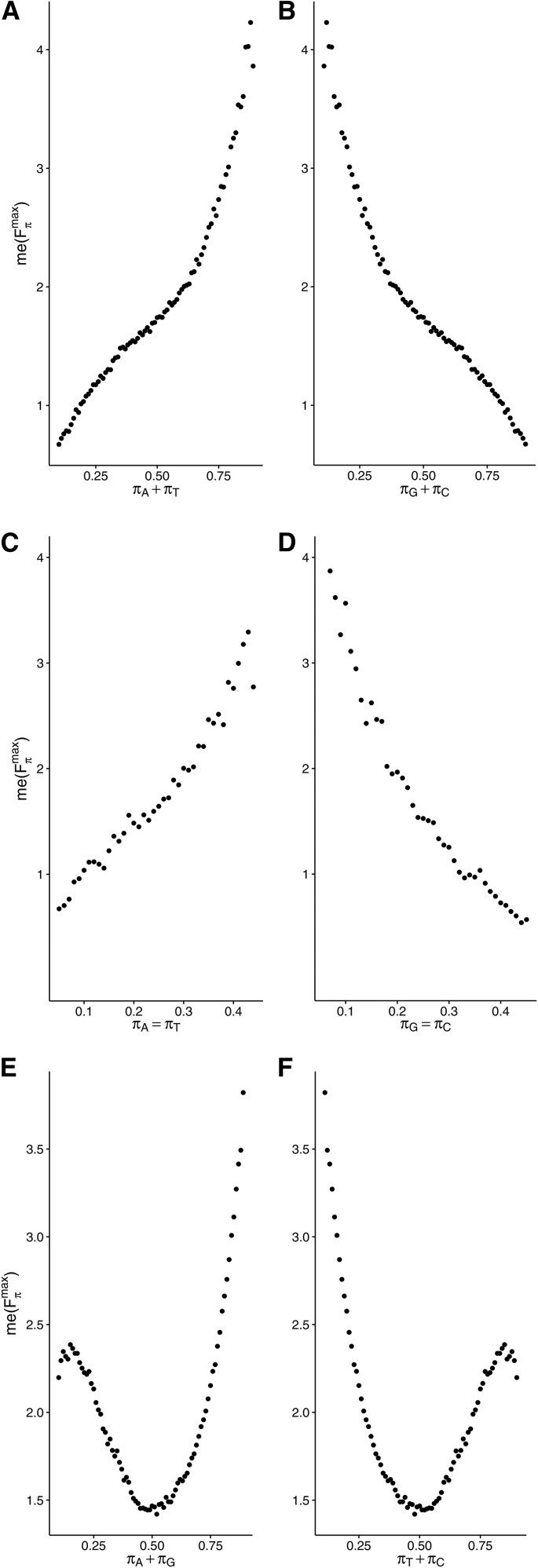
Dependence of median value of $F_{\pi }^{\max},$ *i.e.*, $me\left(F_{\pi }^{\max}\right)$ on stationary content of: adenine + thymine (A), guanine + cytosine (B), adenine and thymine (C), and guanine and cytosine (D) with equal frequencies, as well as purines (E) and pyrimidines (F). There is a clear nonlinear relationship with the minimum for equal proportions of purines and pyrimidines.

Since the median value of $F_{\pi }^{\max}$ depends on the complementary nucleotides in a similar way, we examined the dependence of $me\left(F_{\pi }^{\max}\right)$ on the aggregated frequencies of the nucleotides, A+T and G+C, *i.e.*, $\Pi^{A+T}$ and $\Pi^{G+C}.$ They are both defined in the analogous way. For example, in the case of $\Pi^{A+T}$ we have:

$$\Pi^{A+T}=\underset{k\in K}{\cup }\left\{\pi :\pi \in \Pi \wedge \pi_{A}+\pi_{T}=k\right\},$$

where $\pi_{G}+\pi_{C}=1-\left(\pi_{A}+\pi_{T}\right)$ and k∈K={0.05,0.06,…,0.84,0.85}. In this case, we observed a sigmoidal increase of $me\left(F_{\pi }^{\max}\right)$ with A+T content (Figure 4A), and the opposite trend for G+C (Figure 4B).

The similar dependence of $F_{\pi }^{\max}$ on the frequencies of complementary nucleotides justifies considering also simpler models and stationary distributions, *e.g.*, assuming equal frequencies of the complementary nucleotides: $\pi_{A}=\pi_{T}$ and $\pi_{G}=\pi_{C}.$ This assumption was tested by Morton (2001) on the example of four mutation-selection models. Here, we included a generalization of this model, analyzing a wider range of possible nucleotide frequencies $\pi \in \Pi^{A=T,G=C}.$ The $F_{\pi }^{\max}$ shows an exponential growth from 0.541 (for $\pi_{A}=\pi_{T}=0.06,\pi_{G}=\pi_{C}=0.41$) to 5.010 (for $\pi_{A}=\pi_{T}=0.41,\pi_{G}=\pi_{C}=0.09$), with an increase in the A and T frequency (Figure 4, C and D).

The results show that a strong relationship exists between $F_{\pi }^{\max}$, and the frequencies of complementary nucleotides, regardless of the type of model assumed. Therefore, we can infer that the impact of selection, at the amino acid level, on the usage of 4FD codons is connected with the structure of the stationary distribution generated by its mutation accumulation process. Generally, selection is responsible for the high deviation in the synonymous codon usage when the nucleotide substitution process generates a high frequency of A and T nucleotides, while the processes with a high frequency of G+C in their stationary distributions reduces the impact of selection.

Surprisingly, we observed a completely different dependence of $me\left(F_{\pi }^{\max}\right)$ on the total frequency of purines (A+G) and pyrimidines (C+T) in the assumed stationary distributions, *i.e.*, $\pi \in \Pi^{A+G}$ and $\pi \in \Pi^{C+T}.$ This dependence turned out to be nonlinear and nonmonotonic, in contrast to the complementary nucleotides. In the case of purines, $me\left(F_{\pi }^{\max}\right)$ contains the local maximum at about $\pi_{A}+\pi_{G}=0.13$, and then it drops below 1.5 at about $\pi_{A}+\pi_{G}=0.5,$ reaching the global minimum (Figure 4, E and F). Next, it significantly increases to the global maximum at $\pi_{A}+\pi_{G}=0.9,$ with a value of ∼3.9. The dependence of $me\left(F_{\pi }^{\max}\right)$ on pyrimidines shows a symmetrical course (Figure 4, E and F), with the global maximum at $\pi_{C}+\pi_{T}=0.11,$ the global minimum at $\pi_{C}+\pi_{T}=0.5$, and the local maximum at $\pi_{C}+\pi_{T}=0.9.$

### The effect of selection on particular codon groups

The results presented above referred to the summarized effect of the selection at the amino acid level on all five groups of 4FD codons. However, it is interesting to analyze how selection influences deviation in the expected relative usage of particular groups of the codons *s* for particular nucleotide stationary distributions *π*, *i.e.*, $F_{\pi |s}^{\max}.$The extreme values of $F_{\pi |s}^{\max}$found for the particular codon groups are shown in Table 3. The results demonstrate that the biggest deviation concerns codons coding glycine, whereas the smallest is for valine codons. The other three groups of codons have comparable values. This effect could be explained by differences in the acceptance probabilities of substitutions of amino acids coded by these codon blocks. However, differences between groups of codons disappear in the case of the lowest $F_{\pi }^{\max}$ values, where we observed very similar values of $F_{\pi |s}^{\max}.$

**Table 3:** The highest and the lowest values of $F_{\pi |s}^{max},$ which were found for five 4FD codon blocks denoted by coded amino acids

Selection model	Gly	Val	Thr	Ala	Pro
SM	2.09	1.32	1.84	1.97	1.89
	0.05	0.06	0.06	0.04	0.05
SL	2.09	1.33	1.89	1.99	1.92
	0.05	0.08	0.04	0.04	0.04

Models with the selection assuming lethal substitutions involving stop codons (SL), and the variant with the minimal acceptance probability of such substitutions (SM), were considered separately.

**Figure 5:**
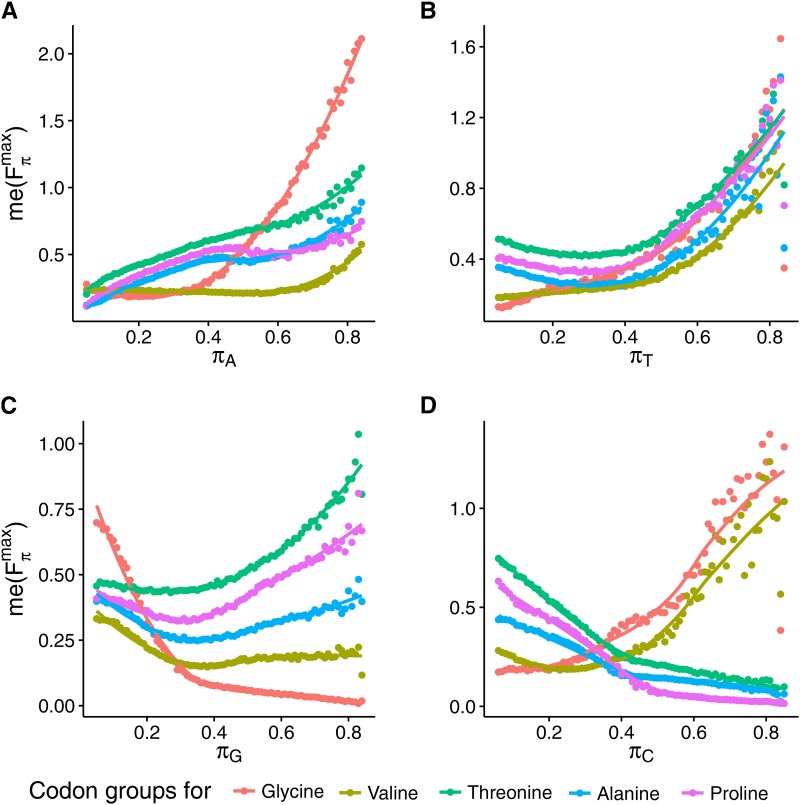
Dependence of the median value of $F_{\pi |S}^{max},$ *i.e.*, $me\left(F_{\pi |S}^{max}\right)$ for 4FD codon groups (assigned by their coded amino acids) on the stationary frequencies of four nucleotides *π*: adenine (A), thymine (B), guanine (C), and cytosine (D). The dots represent exact values of $me\left(F_{\pi }^{max}\right),$ whereas lines are the best approximation based on generalized additive models with integrated smoothness estimation. The median value depends differently on the codon groups and nucleotides.

We additionally tested the deviation in the expected relative usage of particular codon groups $F_{\pi |s}^{\max}$ as a function of stationary nucleotide distribution for the SM model (Figure 5). As in our analysis of the summarized effect on these groups, we likewise calculated the median of $F_{\pi |s}^{\max}$ using the values that were obtained from the substitution matrices generating nucleotide stationary distributions with a fixed frequency of one nucleotide and random frequencies of others. Similarly to the global effect, the same tendency in the case of adenine and thymine was noted. The median of $F_{\pi |s}^{\max}$increases with a comparable intensity for all codon groups as a function of $\pi_{T},$but, in the case of $\pi_{A},$ $me\left(F_{\pi }^{\max}\right)$ for glycine codons grow substantially faster than other codon blocks (Figure 5). The trends are different for the codon blocks depending on guanine and cytosine frequencies. In the case of guanine frequency, $me\left(F_{\pi |s}^{\max}\right)$ decreases substantially for the Gly codon group with $\pi_{G}$ growth, in contrast to the other codon blocks, whose $me\left(F_{\pi |s}^{\max}\right)$ values begin to increase at $\pi_{G}=0.35.$ Two groups of codons can be distinguished when the relationship between $me\left(F_{\pi |s}^{\max}\right)$ and cytosine frequency is taken into account. One group, including the codons for Pro, Ala and Thr, shows a decreasing trend in their $me\left(F_{\pi |s}^{\max}\right),$ whereas the median values of $F_{\pi |s}^{\max}$ of the other group, containing the Gly and Val codons, increase substantially with $\pi_{C}.$

The median of $F_{\pi |s}^{\max}$ for all codon blocks shows a concordant increasing trend with A+T content (Figure 4A), and decreasing for G+C (Figure 4B) for all codons’ groups. The smallest deviation was observed in the codons for valine. As expected, $F_{\pi |s}^{\max}$ also grows for all codon groups with A and T frequencies, with the assumption that $\pi_{A}=\pi_{T}$ and $\pi_{G}=\pi_{C}$ (data not shown).

**Figure 6:**
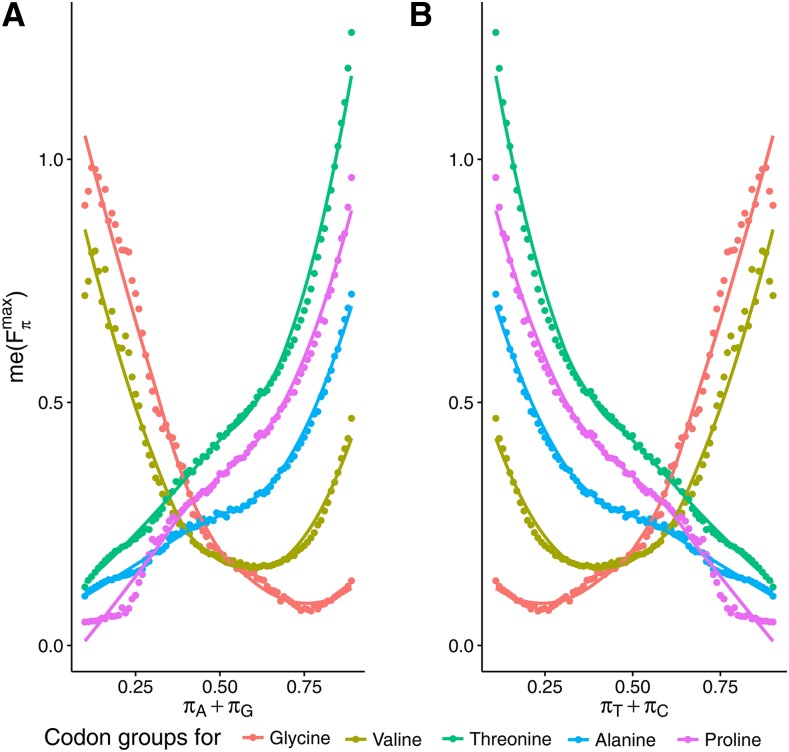
Dependence of the median value of $F_{\pi |S}^{max},$ *i.e.*, $me\left(F_{\pi |S}^{max}\right)$ for 4FD codon groups (assigned by their coded amino acids) on the stationary frequencies of purines (A) and pyrimidines (B). The dots represent exact values of $me\left(F_{\pi }^{max}\right),$ whereas lines are the best approximation based on generalized additive models with integrated smoothness estimation. The groups of codons response differently to the frequencies.

The analysis of $me\left(F_{\pi |s}^{\max}\right)$ for particular codon groups well explains the nonlinear relationship between the summarized effect of selection at the amino acid level on all 4FD codons, and the purines and pyrimidines content (cf. Figure 4, E and F and Figure 6). The median of $F_{\pi |s}^{\max}$ for the Thr, Pro and Ala codons increases with A+G frequency, whereas $me\left(F_{\pi |s}^{\max}\right)$ for the Gly codons decreases. This measure for Val codons also declines with purine content, but reaches its minimum at $\pi_{A}=\pi_{G}=0.6$ and then goes up. The relationships between $me\left(F_{\pi |s}^{\max}\right)$ and C+T content are mirrored. The superposition of these various trends for particular codon groups leads to the nonlinear course of the relationship for the global measure $me\left(F_{\pi }^{\max}\right)$ for all synonymous codons (Figure 4, E and F).

### Characteristics of mutational probability matrices maximizing selection effect

The present study revealed that the effect of selection, at the amino acid level, on synonymous codon usage depends strongly on the nucleotide stationary distributions, which are the result of mutational processes described by substitution probability matrices. Therefore, it is interesting to check what types of nucleotide substitutions are responsible for enhancing this effect. Table 4 presents the best matrix, which, together with selection on nonsynonymous substitutions, maximizes $F_{\pi }.$ The matrix is characterized by the most frequent substitution C→G, and next G→A and C→A, whereas the rarest substitution in the matrix is A→G (Table 4).

**Table 4:** Transition probability matrix $P_{\pi }^{max}$ that maximizes the effect of selection on the relative usage of 4FD codons

	A	T	G	C
A	0.8882	0.0013	0.0000	0.1104
T	0.0008	0.9882	0.0007	0.0103
G	0.1729	0.1549	0.5864	0.0858
C	0.1719	0.0025	0.2884	0.5372

This generates, together with the selection, the largest value of **$F_{\pi }^{max}=9.10$** under SM variant. The stationary distribution of the matrix is: $\pi_{A}=0.19,\;\pi_{T}=0.69,\;\pi_{G}=0.05,\text{ and }\pi_{C}=0.07.$ A nucleotide in the column is substituted by a nucleotide in the row.

To check if these properties are universal, we compared 5% (*i.e.*, 4428) of top matrices that generated the highest values of $F_{\pi }^{\max}$ (shown in Figure S2). The maximizing matrices are characterized by a higher probability of staying the same for adenine and thymine than for guanine and cytosine. Their most frequent substitutions are C→A and C→G. This may result from the fact that these transitions belong to the most regular of all the 120 possible nonsynonymous and single-nucleotide mutations of 4FD codons. Each of them occurs in 16 cases, and they constitute, in total, 27% of the possible substitutions. On the other hand, the lowest probabilities show substitutions A→G and A→T (Figure S2). They are the least frequent mutations of all possible nonsynonymous mutations involving 4FD codons. Each of them applies in only four cases.

Generally, the maximizing matrices have a tendency to generate more adenine and thymine at the expense of guanine and cytosine. These findings correspond well to the relationships observed between $F_{\pi }^{\max}$ and nucleotide stationary distributions, indicating a greater deviation in synonymous codon usage for nucleotide distributions rich in A and T (Figure 3 and Figure 4).

We also noted that the maximizing matrices are characterized by a preponderance of transversions over transitions, which enhances the impact of selection on relative synonymous codon usage. The median and quartile range of transitions to transversions ratio is 0.207 [0.133–0.329]. It is <0.5 when there is no bias toward either transitions or transversions because there are twice as many possible transversions as transitions. This may be attributed to lower acceptance probabilities for amino acid substitutions in Grantham’s (1974) matrix employed in the research, which result from transversions rather than from transitions of the corresponding codons. Actually, the mean acceptance probability for transversions and transitions is 0.409 and 0.538, respectively. The difference is statistically significant in the Mann–Whitney test, with *P* = 0.00001. A higher rate of transversions can, understandably, increase the rare substitutions of codons, and lead to a marked bias in the relative usage of 4FD codons.

The maximizing matrices are also characterized by a significant deviation in the pairs of symmetric nucleotide substitutions, *e.g.*, A→C and C→A expressed by:

$$Dev_{rev}=\sum_{X,Y\in A,T,G,C}\left|p_{X\to Y}-p_{Y\to X}\right|.$$ (13)

Median and quartile range for these matrices were 0.623 [0.491–0.747]. Deviation in pairs of such nucleotide substitutions can enhance the impact of selection on the relative 4FD codon usage through an unbalanced influx and outflow of these codons. For example, C→A substitution, which is more frequent than A→C substitution, can lower the content of alanine codon GCC at the expense of GAC coding for asparagine. In the case of comparable probabilities of these substitutions, reversions could recover the numbers of disappearing codons.

### Comparison of estimated deviations in codon usage with that observed in protein coding genes

It would be desirable to assess the strength of the measured effect of selection, at the amino acid level, on the relative usage of 4FD codons in the context of empirical data. Therefore, we compared the difference observed between the relative frequencies of 4FD codons after selection, and their expected frequencies resulting only from the applied mutational process, with an analogous measure for such codons calculated in protein coding sequences present in almost 4900 bacterial genomes. In an ideal situation, the expected occurrence of the observed relative frequencies of 4FD codons in protein coding sequences should be an aftermath of pure mutational pressures only. These are, however, not known. Therefore, we approximated the expected frequencies of 4FD codons by the average of the relative frequencies of 4FD codons in genes. Since bacterial genomes are characterized by a strong chromosome-wide compositional bias determined by two mutational pressures associated with differently replicated, leading and lagging, DNA strands, we examined the codon usage of genes separately from these DNA strands.

**Figure 7:**
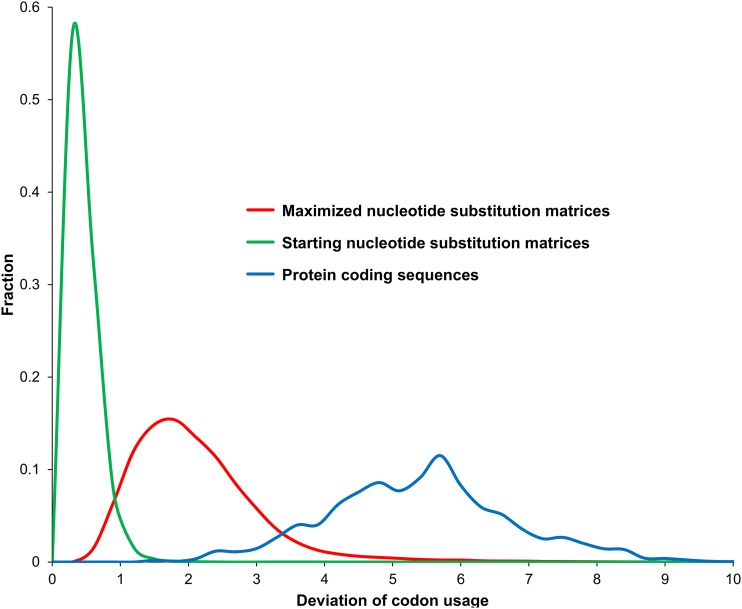
Distribution of the deviation from the expectation in the codon usage for all 4FD groups calculated for protein coding sequences, starting (randomly selected) nucleotide substitution matrices, and matrices that maximized this measure. The maximized values are of the same order of magnitude as the deviation based on empirical data.

The distribution of the summarized deviation from the expectation distribution in codon usage for all 4FD groups in protein-coding sequences was compared with the distributions of the calculated maximum deviation in codon usage resulting from selection at the amino acid level (the measure $F_{\pi }^{\max}$). It was also compared with the starting values before the optimization procedure (Figure 7). The maximized values are clearly shifted from the initial distribution, and overlap the distribution from genes. The average figure for the starting values is 0.3, for the maximized values ∼2, whereas for the genes it is 5.2. The figures for the maximized nucleotide substitution matrices constitute, on average, 37% of the deviation in codon usage found for protein coding sequences.

The data seem to indicate that the estimated effect of the selection by the measure $F_{\pi }^{\max}$ is not negligible. It is likely that the deviation calculated for the real genes is higher because of additional factors influencing codon bias, and linked, for example, with the effectiveness of translation, which appears universal in prokaryotic genomes, and concerns a substantial fraction of their genes (Supek *et al.* 2010). It is conceivable that the applied selection model based on the general Grantham’s (1974) amino acid matrix deviates from synonymous codon usage less than in real selections, which can be different for various genes and their products. Nevertheless, comparison with the observed data demonstrates that the effect of selection at the amino acid level could help explain a substantial proportion of the observed codon usage bias, and, as such, cannot be disregarded.

### Modeling of codon substitution process

In this study, we used a mutational–selection model the same as that proposed by Morton (2001). This approach has many advantages that are relevant to our study. The model consists of separate mutation and selection components, which are easy to control. The mutation process can be defined simply by mutational matrices based on fixed nucleotide stationary distributions without any influence of selection. Apart from stationarity, the mutational matrix does not require the assumption on time-reversibility, which makes this model more general. The selection part is also expressed easily by an amino acid matrix, which does not require complicated transformations and implementations.

We decided to apply this model because others commonly used in the modeling of codon substitutions (Halpern and Bruno 1998; Yang and Nielsen 2008) are not flexible enough to investigate the studied phenomenon. Particularly, they describe the mutation substitutions as a time-reversible stationary Markov process. They also introduce a selection mechanism in such a way that the final mutation-selection process is also time-reversible and stationary. Thanks to that, the models are computationally effective tools. However, there are no biological reasons to expect that the substitution process proceeds in a reversible manner (Felsenstein 2004; Schneider and Cannarozzi 2012; Yang 2006). This assumption is used only because of theoretical and practical computational benefits, as well as mathematical convenience.

It is worth pointing out that, when we implement, in these models, a selection based on amino acid frequencies determined by functional requirements in proteins, then these models will produce the relative stationary frequencies of synonymous codons that will be the same as the stationary frequencies resulting from the strict mutational process (Wallace *et al.* 2013; Yang and Nielsen 2008). In consequence, under these assumptions, it is impossible to investigate the impact on synonymous codon usage of selection at the amino acid level. Therefore, the model applied in this work appears to be more elastic and general because of less restrictive assumptions. In addition, it does not exclude “*a priori*” any possible additional factors that could influence the usage of synonymous codons.

The time-reversibility assumption is crucial in our consideration because if we assume that the nucleotide substitution process defined by the matrix P* is time-reversible, and the acceptance matrix *D* is symmetric, then the impact of selection at the amino acid level on the usage of synonymous codons disappears. To prove this, it is enough to show that, under the above assumptions, the combined mutation–selection process defined by the probability matrix *C* has the same stationary distribution as the mutational process alone, *i.e.*, $\pi^{sel}=\pi^{cod}.$ Thus, from the time-reversibility, we get:

$$p_{k\to l}^{*}\times \pi_{k}^{cod}=p_{l\to k}^{*}\times \pi_{l}^{cod}$$ (14)

and assuming the symmetry of the acceptance matrix *D*, *i.e.*, $d_{m\to n}=d_{n\to m},$ we obtain the following equalities:

$$c_{k\to l}\times \pi_{k}{}^{cod}=p_{k\to l}^{*}\times d_{m\to n}\times \pi_{k}{}^{cod}=p_{l\to k}^{*}\times d_{n\to m}\times \pi_{l}{}^{cod}=c_{l\to k}\times \pi_{k}{}^{cod},$$ (15)

Clearly, Equation 15 is a detailed balance equation of the process generated by the mutation–selection matrix *C*. As a result, this process is also time-reversible, and, consequently, $\pi^{sel}=\pi^{cod}.$ Thus, the studied influence of selection at the amino acid level on synonymous codons usage cannot be demonstrated under the assumption of time-reversibility of the mutational process and the symmetry of the acceptance matrix *D*.

Nevertheless, this property (15) can be used in validation of the searching algorithm applied in our study. Since this algorithm considers the general class of nucleotide mutational matrices, including also the time-reversible models as a subset, it should be possible to find, by this algorithm, such nucleotide transition probability matrices that would generate the exact equality between stationary codon distributions before and after selection, *i.e.*, $\pi^{sel}=\pi^{cod}.$ Such results would imply that the algorithm works efficiently. As expected, we received this equality for nucleotide substitution matrices that minimized the objective function *F_π_*. The average values of *F_π_* were, in these simulations, almost equal to zero for all considered nucleotide stationary distributions.

It should also be added that, if the deviation in synonymous codon usage is obtained under the time-reversible nucleotide substitution matrices, then the acceptance probabilities matrix must be asymmetric. However, commonly used matrices describing physicochemical or biochemical differences/similarities between amino acids are symmetric. In our approach, we applied Grantham’s (1974) matrix of acceptance probabilities corresponding to chemical similarities between amino acids. Since the matrix is symmetric, it favors no direction of amino acid replacement, in contrast to a mutational matrix. Therefore, both mutation and selection are necessary to generate bias in the usage of 4FD codons.

In our model, it is also possible to apply other acceptance probability matrices based on various physicochemical or biochemical amino acid properties, *e.g.*, hydropathy or polarity. Since such properties, and resulting matrices, are usually quite strongly correlated, their use would not change the general conclusion about the influence of selection at the amino acid level on synonymous codon usage. The other matrices can slightly increase or decrease the codon bias observed for Grantham’s (1974) matrix, depending on the stationary distributions of mutational matrices, but comprehensive and detailed studied are necessary to assess these relationships, and the intensity of this effect. However, commonly used PAM (Point Accepted Mutation) matrices are not appropriate because they are not free of a mutational influence, which is important in our considerations. It should also be noted that Grantham’s (1974) matrix represents a mean field approximation of a model with fluctuating selection, because this matrix describes only general similarities in chemical properties of amino acids, but not specific selection for a particular protein. Various types of proteins can be characterized by different selection requirements because of their specific structure and function. The Grantham’s (1974) matrix is a general representation of constant selection, but may not be a good approximation to the true evolutionary dynamics under time-varying selection. Such variable selections can produce their own distinctive pattern in codon usage bias in different types of sequences or regions (Plotkin and Dushoff 2003; Plotkin *et al.* 2006).

### Properties of $F_{\pi }$ measure

To assess the strength of selection at the amino acid level on 4FD codon usage, we applied the $F_{\pi }$ measure, being the normalized difference between the relative frequency of 4FD codons before and after selection (Equation 9 and Equation 10). This measure was inspired by chi-squared statistics, and has useful features, like other standard measures, which can also be applied in the calculation of differences between probability distributions. Above all, $F_{\pi }$ is always non-negative and equals zero if, and only if, the relative stationary frequency of a codon subjected to a mutational process equals its frequency after selection, *i.e.*, πi=πsisel/πssel for all i∈A,T,G,C. This property is called the identity condition, and should be fulfilled by any measure that is used to calculate the difference between two probability distributions. Thanks to that, the detection of any differences between stationary frequencies of π and $\pi_{s}^{sel}$ is independent of the measure. In contrast to standard measures like Kullback-Leibler divergence, or total variation distance, $F_{\pi }$ includes information about the absolute value of the change of codon frequencies in relation to expectation under the mutational process, which is useful to easily interpret the results obtained.

Nevertheless, our measure gives values compatible with Kullback-Leibler divergence and total variation distance. The Spearman correlation coefficient between values of our measure with those of Kullback-Leibler divergence and the total variation distance calculated for all 88,560 considered cases of nucleotide stationary distributions is very high and statistically significant (*p*-values <2.2E−16, 0.921 and 0.915, respectively). Therefore, the application of these measures would change neither conclusions nor important results. With such great correlation, the trends presented in figures between the measure and nucleotide composition would be also the same.

### Concluding remarks

The study undertaken here estimated the influence of selection, at the amino acid level, on the relative usage of 4FD codons. This impact was determined by different selection constraints on the nonsynonymous replacement of these codons with others, which proceeded in a complex manner and depended on the probability of fixation of such substitutions, as well as on the probability of particular nucleotide substitutions. We tested a wide range of conditions in which such influence can be valid, by the inclusion of nearly 90,000 stationary nucleotide distributions and associated unrestricted mutational processes. Selection was based on differences in the physicochemical properties of amino acids.

We noticed that mutational processes generating more adenine and thymine than guanine and cytosine enhance the influence of selection. The same is true for the processes yielding more purines than pyrimidines. It is noteworthy that the relationship between the effect under study and the content of these nucleotides is nonlinear. On the other hand, the impact of selection at the amino acid level diminishes when the nucleotide processes generate 50% content of purines and pyrimidines as well as more guanine and cytosine than adenine and thymine. The nucleotide substitution matrices maximizing the consequence of amino acid selection are also characterized by a greater probability of transversions outnumbering transitions, and a greater deviation in pairs of reversible nucleotide substitutions.

The influence of selection at the amino acid level was different for particular groups of 4FD codons. Generally, glycine codons show the strongest response to the selection impact under study, whereas codons for valine the weakest. However, the deviation in the codon usage generated by the process, with and without selection, depends nonlinearly on nucleotide stationary distribution. This effect could be explained by the discrepancies in the acceptance probabilities of substitutions of amino acids coded by these codon blocks.

The results indicate that selection acting on nonsynonymous substitutions, *i.e.*, leading to amino acid replacements, can affect the usage of 4FD codons. This effect, however, is complex, and depends on the properties of mutational pressure, which superimposes on the selection. Interestingly, we discovered that, for each nucleotide distribution, it is possible to find such mutational probability matrices that will minimize and maximize the effect. This seems to suggest that the influence of selection, at the amino acid level, on synonymous codon usage, can vary in different organisms. Since the mutational pressure in genomes is not known, and selection at the amino acid level is also complicated, it is difficult to assess the exact contribution of this process in real protein coding sequences. Selection can both enhance and suppress the other effects on codon usage, *e.g.*, selection related to translation efficiency. Nevertheless, the effect cannot be neglected because it correlates with the comparison between the calculated deviation in the codon usage subjected to this selection and an analogous measure estimated for protein coding sequences.

**Figure 8:**
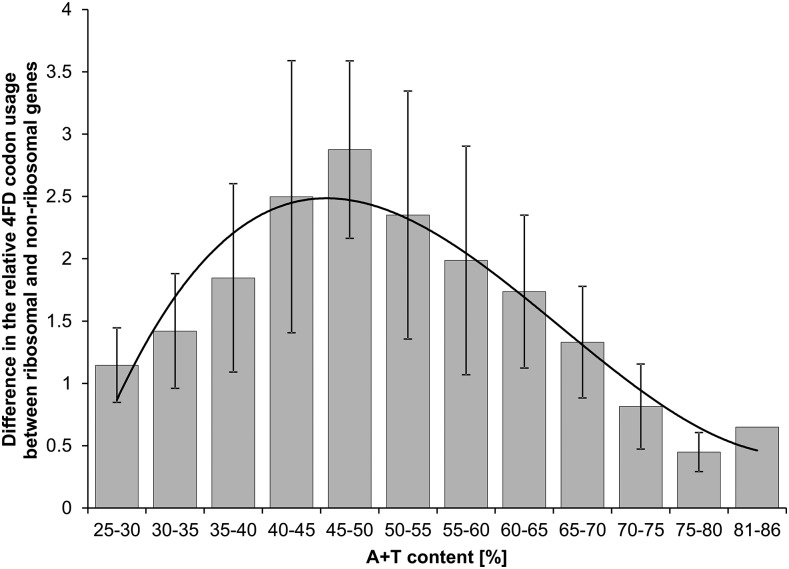
Dependence on the genomic A+T content of the difference in the relative usage of 4FD codons between genes coding for ribosomal and nonribosomal proteins. The difference was calculated based on 4802 genomes, with at least 30 genes annotated for ribosomal proteins, separately for the leading and lagging strand. In total, 5124 pairs of genes, with at least 15 ribosomal genes on one strand, were considered. The bars represent an average value for the given class of A+T content, whereas whiskers represent SD. The difference was calculated according to: $\sum_{s\in S}\sum_{i\in A,T,G,C}\left|o_{s_{i}}^{rib}/o_{s}^{rib}-o_{s_{i}}^{nonrib}/o_{s}^{nonrib}\right|,$ where $o_{s_{i}}^{}$is the observed frequency of a 4FD codon *s_i_* with a nucleotide *i* at the third codon position, and $o_{s}^{}=\sum_{i\in A,T,G,C}o_{s_{i}}^{}$ is the frequency of all codons in the 4FD codon group *S*. Indices *rib* and *nonrib* mean genes for ribosomal and nonribosomal proteins, respectively. The calculated difference decreases with AT%, and is the largest for the moderate AT content.

Our results show that substitution matrices generating high A+T content affect 4FD codon usage to the greatest extent. Assuming that the global genome content corresponds to a global mutational pressure (Muto and Osawa 1987), we can conclude that the effect of the selection under study would be most pronounced in AT-rich genomes. Consequently, the selection on nonsynonymous substitutions can interfere in such genomes with other selections on codon usage, *e.g.*, related to translational efficiency. In agreement with that, we found that the difference between highly expressed genes coding for ribosomal proteins and other genes, as far as the relative usage of particular 4FD codons is concerned, becomes smaller with A+T genomic content (Figure 8). There seems to be some evidence that it could be more difficult to maintain the appropriate codon bias in highly expressed genes in AT-rich genomes. Likewise, in genomes with >70% A+T, no influence of translational selection was reported, *i.e.*, *Borrelia burgdorferi* (McInerney 1998), *Buchnera* (Rispe *et al.* 2004), *Wigglesworthia* (Herbeck *et al.* 2003) and *Blochmannia floridanus* (Banerjee *et al.* 2004). This may result from a greater difficulty in predicting genes with translational efficiency in AT-biased genomes using standard methods (*e.g.*, CAI, codon adaptation index), because other methods based on random forest classifier revealed that, in these genomes, codon bias was associated with translational efficiency (Supek *et al.* 2010). Notwithstanding, our results show that AT-rich genomes either have to cope with the greater influence of selection, at the amino acid level, on synonymous codon usage or adapt to it. It is possible that this type of selection can trigger codon bias in some genes, which can be misleading with regard to selection on translational effectiveness. Accordingly, Morton (2001) carried out an appropriate test, decreasing the number of genes believed to have codon usage associated with translational selection.

The aim of our study was to verify the effect of amino acid selection on 4FD codon usage on global and general scales for a large number of possible mutational pressures, and fixed selection for amino acid replacements. Nevertheless, our results can be helpful to explain some effects related to codon bias also at the local scale, *i.e.*, codon usage variation across sites within a gene. Such variation was noted by Akashi (1994) in orthologous genes from fruit flies. He found that the frequency of preferred codons is significantly higher at conserved amino acid positions than in nonconserved ones. This finding was further confirmed in bacteria (Stoletzki and Eyre-Walker 2007), as well as yeast, worm, mouse, and human (Drummond and Wilke 2008). This codon bias was interpreted as a result of selection for minimization of the chance for translation errors and protein misfolding during this process. On the other hand, Plotkin and Dushoff (2003) and Plotkin *et al.* (2004) found that some variable sequences coding for antigens and surface proteins, or regions interacting with antibodies in pathogens *Mycobacterium tuberculosis*, *Plasmodium falciparum*, and influenza A virus, are rich in “volatile” codons that can mutate with larger probability to codons encoding other amino acids. Such elevated volatility of these genes may be associated with a positive selection, and greater pressure for amino-acid substitutions, which is favored in order to avoid interactions with the host immune system. Although we showed that a synonymous codon bias can be generated by a general selection at the amino acid level, it cannot be excluded that more specific selections influencing particular sites in protein sequences with various intensity or pattern (Bazykin 2015) may also contribute, with other effects, to the observed codon biases at specific sequence sites.

## Supplementary Material

Supplemental material is available online at www.g3journal.org/lookup/suppl/doi:10.1534/g3.116.038125/-/DC1.
